# Breastmilk transfer and infant exposure to atazanavir/ritonavir in breastfeeding women with HIV: results from IMPAACT 2026

**DOI:** 10.3389/frph.2026.1892081

**Published:** 2026-08-21

**Authors:** Emily Anne Barr, Mauricio Pinilla, Jeremiah D. Momper, Ahizechukwu C. Eke, Lucy Koech, Kristina M. Brooks, Victoria Ndyanabangi, Sandra K. Burchett, Sujith Valiyaparambil, Mark Mirochnick, Alice Marie Stek, Brookie M. Best, Rachael Jeffrey, Chelsea Stotz, Marije Van Schalkwyk, Renee Browning, David E. Shapiro, Kathleen M. Powis

**Affiliations:** 1 Department of Research, University at Buffalo School of Nursing, Buffalo, NY, United States; 2 Cizik School of Nursing, University of Texas Health Science Center at Houston, Houston, TX, United States; 3 Center for Biostatistics in AIDS Research, Harvard T.H. Chan School of Public Health, Boston, MA, United States; 4 Skaggs School of Pharmacy and Pharmaceutical Sciences, University of California San Diego, La Jolla, CA, United States; 5 Division of Maternal-Fetal Medicine, Department of Gynecology and Obstetrics, Johns Hopkins University School of Medicine, Baltimore, MD, United States; 6 Walter Reed Project Clinical Research Center, Kenya Medical Research Institute (KEMRI), Kericho, Kenya; 7 Department of Pharmaceutical Sciences, Skaggs School of Pharmacy and Pharmaceutical Sciences, University of Colorado Anschutz, Aurora, CO, United States; 8 Baylor College of Medicine Children's Foundation Uganda, Kampala, Uganda; 9 Division of Infectious Diseases, Department of Medicine, Boston Children's Hospital and Harvard Medical School, Boston, MA, United States; 10 Laboratory Data Management, Frontier Science Foundation, Amherst, NY, United States; 11 Department of Pediatrics, Boston University Chobanian & Avedisian School of Medicine, Boston, MA, United States; 12 Boston Medical Center, Boston, MA, United States; 13 Division of Maternal-Fetal Medicine, Department of Obstetrics and Gynecology, Keck School of Medicine, University of Southern California, Los Angeles, CA, United States; 14 Department of Pediatrics, School of Medicine, University of California, San Diego, CA, United States; 15 FHI 360, Durham, NC, United States; 16 IMPAACT Clinical Data Management, Frontier Science Foundation, Amherst, NY, United States; 17 Family Centre for Research with Ubuntu, Division of Adult Infectious Diseases, Department of Medicine, Stellenbosch University and Tygerberg Hospital, Cape Town, South Africa; 18 National Institute of Allergy and Infectious Diseases, National Institutes of Health, Bethesda, MD, United States; 19 Departments of Internal Medicine and Pediatrics, Massachusetts General Hospital, Boston, MA, United States; 20 Department of Immunology and Infectious Diseases, Harvard T.H. Chan School of Public Health, Boston, MA, United States

**Keywords:** antiretrovirals, atazanavir, breastfeeding, breastmilk, HIV, pharmacokinetics, relative infant dose, ritonavir

## Abstract

**Background:**

Breastfeeding offers significant health benefits, and guidance for women with HIV increasingly emphasizes individualized, informed infant-feeding decisions. Breastmilk antiretroviral transfer and safety data are needed to inform shared decision-making discussions across all settings.

**Methods:**

Breastmilk transfer of ritonavir (RTV)-boosted atazanavir (ATV) was evaluated in the International Maternal Pediatric Adolescent AIDS Clinical Trials (IMPAACT) 2026 study. Fourteen breastfeeding women with HIV receiving RTV boosted ATV 100/300 mg daily, as part of a multi-drug regimen, and their infants were enrolled in Kenya, Uganda, and South Africa between 5 and 9 days postpartum and followed through age 16–24 weeks. Paired maternal plasma, breastmilk, and infant plasma samples were collected at each visit. Concentrations were measured using validated LC-MS/MS methods. Relative infant dose (RID) was estimated assuming an infant milk intake of 150 mL/kg/day. Safety outcomes were prospectively assessed.

**Results:**

All 14 women reported exclusive breastfeeding. Median maternal plasma and breastmilk concentrations were 1,410 ng/mL and 432 ng/mL for ATV, and 217 ng/mL and 13.6 ng/mL for RTV, respectively. Median breastmilk-to-plasma ratios were 0.253 (ATV) and 0.081 (RTV). RID values were 1.27% (maximum 6.43%) for ATV and 0.12% (maximum 1.10%) for RTV. Infant plasma concentrations were below the lower limit of quantitation (ATV 23.4 ng/mL; RTV 9.8 ng/mL) in all 42 samples. No maternal adverse events occurred; three infants experienced unrelated grade 2 events.

**Conclusions:**

In this cohort, ATV and RTV were detectable in maternal plasma and breastmilk but not in infant plasma, with low RID estimates and a favorable safety profile.

## Background

The incidence of perinatal HIV acquisition has been significantly reduced over the past decade in the maturing HIV epidemic. This is due, in part, to more potent and better tolerated antiretroviral drugs available for use in pregnancy and postpartum, as well as policy changes that promote universal access to HIV treatment at time of diagnosis (1–3). Among postpartum women with HIV who opt to breastfeed, increased access to antiretroviral treatment (ART) has significantly reduced infant HIV acquisition (4–8). In high HIV prevalence settings, breastfeeding has been recommended because of its substantial maternal and infant health benefits, including nutritional, immunological, and developmental advantages (9–16). However, historically in high income countries, guidelines exclusively promoted formula feeding for infants born to women with HIV, to avoid any possible risk of HIV transmission inherent in breastfeeding (4, 17–19). Yet with more potent ART options safe for use in pregnancy and postpartum, the risk-benefit trade-off between vertical transmission and health benefits of breastfeeding for both the mother and infant have shifted (20, 21).

Recognizing this evolution, guidelines have been updated, including those from the U.S. Department of Health and Human Services (7) and the American Academy of Pediatrics (4), largely driven by advocacy from people with HIV and community-based organizations. The revised policy recommendations reflect an approach that calls for shared decision-making between health providers and pregnant and postpartum women and their partners. This includes individualized counseling for women with HIV who desire to breastfeed with continued support and discussions throughout the breastfeeding period. These guidelines acknowledge that with sustained viral suppression, the risk of HIV transmission via breastfeeding is less than 1%, but not zero (7) and provide an opportunity for informed choice in infant feeding options among parents with HIV (20, 22).

Clinical lactation studies of antiretroviral medications taken by women who are breastfeeding are essential to understand breastmilk drug transfer to the infant and its safety implications (23, 24). While some antiretrovirals, such as zidovudine and lamivudine, have demonstrated high transfer rates through breastmilk, others, like lopinavir, a protease inhibitor, exhibit lower concentrations in breastmilk, reducing concerns about infant toxicity (25, 26). Understanding the kinetics of breastmilk transfer of newer protease inhibitors, such as atazanavir/ritonavir (ATV/r), is necessary for its safe use during lactation, but currently available data are limited (27, 28).

In this arm of the International Maternal Pediatric Adolescent AIDS Clinical Trials (IMPAACT) 2026 study, we sought to quantify concentrations of ATV and RTV in breastmilk, systemic concentrations in breastfed infants, and overall safety of infant exposure to ATV and RTV.

## Methods

### Study population and design

The IMPAACT 2026 study is a nonrandomized, open-label, multi-center phase-IV prospective study of the pharmacokinetics (PK) and safety of selected antiretroviral and anti-tuberculosis medications used during pregnancy and postpartum (ClinicalTrials.gov NCT04518228). This analysis focuses on postpartum women living with HIV who received RTV-boosted ATV 100 mg/300 mg once daily in a multi-drug regimen as part of their routine clinical care. IMPAACT 2026's opportunistic study design allowed for enrollment of individuals already prescribed ART by their clinical providers; the study team did not initiate, modify, or influence any aspect of antiretroviral treatment decisions. Participants were recruited at study sites in Kenya, Uganda, and South Africa where RTV-boosted ATV was routinely used as part of clinical HIV care. The protocol was approved by all required institutional review boards, ethics committees, and applicable regulatory entities at all participating sites. All maternal participants provided written informed consent on their own behalf and on behalf of their infants.

Postpartum women were able to enroll provided they met the following eligibility criteria: a confirmed diagnosis of HIV and were breastfeeding while receiving RTV boosted ATV for at least two weeks before enrollment and with plans to continue the current regimen through at least 16 weeks postpartum. Participants were required to meet additional inclusion criteria, including having a stable clinical condition and no severe maternal or infant medical conditions that would make infant survival beyond 24 weeks unlikely. Mother-infant pairs were enrolled between 5 and 9 days postpartum and followed until 16–24 weeks postpartum or cessation of breastfeeding, whichever occurred first. The study aimed to enroll up to 15 mother-infant pairs with evaluable PK and safety data.

### PK sampling and drug concentration measurements

Samples for the quantification of ATV and RTV concentrations were collected at three postpartum visits: enrollment (5–9 days postpartum), 2–12 weeks postpartum, and 16–24 weeks postpartum. At each visit, maternal plasma, breastmilk, and infant plasma samples were collected according to IMPAACT 2026 breastmilk transfer pharmacokinetic procedures. Maternal plasma, breastmilk, and infant plasma samples were obtained within a 90-minute sampling window to support paired maternal-infant analyses. Participants were required to be actively breastfeeding and receiving the antiretroviral regimen under study at the time of sampling. Breastmilk was collected via hand expression or pump. Whole breastmilk was analyzed without separation of milk components. ATV and RTV concentrations in plasma and whole breastmilk were measured using validated liquid chromatography-tandem mass spectrometry (LC-MS/MS) methods. Prior to LC-MS/MS analysis, plasma and whole breastmilk samples underwent protein precipitation with acetonitrile to precipitate proteins and reduce matrix-related interferences, including those associated with the higher protein and lipid content of breastmilk. Method validation in both matrices demonstrated acceptable matrix effect, recovery, and process efficiency, supporting the suitability of the assay for quantification of ATV and RTV in plasma and whole breastmilk. Matrix-specific lower limits of quantitation (LLoQ) were established for plasma and whole breastmilk specimens. The LLoQ for ATV was 23.4 ng/mL for plasma and 3.91 ng/mL for breastmilk. The LLoQ for RTV was 9.8 ng/mL for plasma and 0.977 ng/mL for breastmilk.

### Safety assessments

Safety data were collected for both maternal and infant participants prospectively throughout the study. Adverse events (AEs) were graded using the Division of AIDS (DAIDS) Table for Grading the Severity of Adult and Pediatric Adverse Events [version 2.1, July 2017; National Institute of Allergy and Infectious Diseases (29)] and were evaluated by site investigators for severity and relatedness to ATV or RTV. Infant HIV infection status was assessed according to local standard-of-care diagnostic procedures during study participation. Maternal safety evaluations included clinical monitoring for AEs and documentation of concomitant medications, which were reviewed to assess whether events could be attributed to ATV, RTV, or to other causes. Infant safety assessments included monitoring for AEs potentially associated with ATV or RTV exposure via breastmilk.

### Statistical analyses

Descriptive statistics were employed to summarize maternal and infant characteristics, PK data, and safety outcomes. Continuous variables were summarized using medians, lower and upper quartiles (Q1, Q3), and the minimum and maximum values, while categorical variables were presented as counts and percentages. In calculating percentages, the denominator reflects the number of participants with data available. ATV and RTV concentrations in maternal plasma, breastmilk, and infant plasma were summarized descriptively and maternal breastmilk/plasma concentration ratios were derived for each visit. PK concentrations from all available visits were summarized descriptively, and overall medians represent pooled observations across all postpartum timepoints. AEs were categorized by severity and potential relatedness to either ATV or RTV exposure. For ATV concentrations below the LLoQ, analyses imputed values of 11.7 ng/mL and 1.96 ng/mL (half the LLoQ) for plasma and breastmilk, respectively. For RTV concentrations below the LLoQ, analyses imputed values of 4.9 ng/mL and 0.488 ng/mL (half the LLoQ) for plasma and breastmilk, respectively. Relative infant dose (RID) was calculated as [infant dose [mg/kg/day] ÷ maternal dose [mg/kg/day]] × 100, assuming an infant milk intake of 150 mL/kg/day, as described previously (30). All analyses were conducted using SAS version 9.4 (SAS Institute Inc., Cary, NC, USA).

## Results

Between May 2022 and November 2023, 14 mother-infant pairs were enrolled in the ATV and RTV arm of the IMPAACT 2026 study. All mothers identified as Black and not of Hispanic or Latina ethnicity. Participants were enrolled in Kenya (*n* = 10, 71%), Uganda (*n* = 3, 21%) and South Africa (*n* = 1, 7%). The median age of maternal participants at study entry was 29.6 years (Q1, Q3: 23.0, 34.3), and the median duration of follow-up postpartum was 20.2 weeks (Q1, Q3: 18.7, 21.6) (Table 1). Infant characteristics at birth included a median gestational age of 40.1 weeks (Q1, Q3: 37.0, 41.6), with 3 (21%) born before 37 weeks. The median birth weight was 3,150 g (Q1, Q3: 2,800, 3,500), with 93% of infants (*n* = 13) weighing ≥2,500 g (Table 1).

**Table 1:** Maternal and infant characteristics at enrollment (*N* = 14 mother–infant pairs).

Characteristic	Median (Q1, Q3) or *n* (%)	Range
Maternal age at entry (years)	29.6 (23.0, 34.3)	19.0–39.4
Race/Ethnicity		
Black, not Hispanic/Latina	14 (100%)	
Country of enrollment		
Kenya	10 (71%)	
Uganda	3 (21%)	
South Africa	1 (7%)	
Maternal-infant pair follow-up time (weeks)	20.2 (18.7, 21.6)	15.3–21.9
Gestational age at delivery (weeks)	40.1 (37.0, 41.6)	34.0–44.4
<37 weeks	3 (21%)	
Birth weight (g)	3,150 (2,800, 3,500)	2,000–4,000
≥2,500 g	13 (93%)	
Birth length (cm)	49.5 (48.5, 50.3)	43.0–52.0

Data are presented as median (Q1, Q3) unless otherwise indicated. Percentages are based on available data.

Feeding practices were consistent throughout the study, with all infants exclusively breastfed. No formula or non-maternal breastmilk was reported at any visit. The median duration of breastfeeding was 20.2 weeks (Q1, Q3: 18.7, 21.6). Thirteen of fourteen infants received nevirapine-based prophylaxis, consistent with standard of care in participating regions (Table 2). Antiretroviral prophylaxis information was not available for the fourteenth infant.

**Table 2:** Infant feeding practices, prophylaxis, and HIV outcomes (*N* = 14 infants).

Characteristic	Median (Q1, Q3) or *n* (%)	Range
Feeding type		
Exclusive breastfeeding	14 (100%)	
Duration of breastfeeding (weeks)	20.2 (18.7, 21.6)	15.3–21.9
Infant prophylaxis		
Nevirapine-based prophylaxis	13 (93%)	
Data unavailable	1 (7%)	
Infant HIV status		
HIV-negative	13 (93%)	
No test result available	1 (7%)	

All infants received exclusive breastfeeding throughout the study. Percentages are based on available data.

Among all maternal plasma samples (*N* = 41), median ATV and RTV concentrations were 1,410 ng/mL (Q1, Q3: 179, 2,510) and 217 ng/mL (Q1, Q3: 4.9, 400), respectively, with a median time of 16.9 (Q1, Q3: 14.3, 18.5) hours between dose and sample collection. Corresponding median breastmilk concentrations were 432 ng/mL (Q1, Q3: 126, 633) for ATV and 13.6 ng/mL (Q1, Q3: 2.12, 24.3) for RTV, with a median time of 17.2 (Q1, Q3: 14.5, 18.9) hours between dose and sample collection. The breastmilk-to-plasma concentration ratios were 0.253 (Q1, Q3: 0.209, 0.369) for ATV and 0.081 (Q1, Q3: 0.051, 0.100) for RTV (Table 3). Breastmilk and plasma concentrations demonstrated interparticipant variability across study visits. Infant plasma concentrations for both ATV and RTV were below LLoQ in all 42 infant samples (14 infants×3 visits). The median RID was 1.27% (maximum 6.43%) for ATV and 0.12% (maximum 1.10%) for RTV (Table 4).

**Table 3:** Maternal plasma and breastmilk atazanavir (ATV) and ritonavir (RTV) concentrations and breastmilk: plasma ratios.

Analyte	Timepoint	*n*	Median (Q1, Q3)	Range	*n* (%) ≥LLoQ
Plasma ATV (ng/mL)	5–9 days	13	1,870 (747, 2,620)	11.7–5,670	10 (77%)
	2–12 weeks	14	836 (149, 2,210)	11.7–4,760	11 (79%)
	16–24 weeks	14	1,620 (1,090, 2,510)	11.7–7,080	12 (86%)
Breastmilk ATV (ng/mL)	5–9 days	14	464 (375, 624)	1.96–1,240	11 (79%)
	2–12 weeks	14	364 (47.3, 550)	1.96–2,190	12 (86%)
	16–24 weeks	14	493 (195, 974)	1.96–2,050	12 (86%)
Breastmilk: Plasma ATV ratio	Overall	41	0.253 (0.209, 0.369)	0.143–44.2^a^	
Plasma RTV (ng/mL)	5–9 days	13	128 (39.1, 419)	4.9–1,600	10 (77%)
	2–12 weeks	14	86.6 (4.9, 339)	4.9–1,900	9 (64%)
	16–24 weeks	14	267 (128, 400)	4.9–2,230	11 (79%)
Breastmilk RTV (ng/mL)	5–9 days	14	13.5 (4.70, 27.5)	0.488–77.9	11 (79%)
	2–12 weeks	14	8.98 (1.13, 24.3)	0.488–125	12 (86%)
	16–24 weeks	14	18.0 (6.20, 22.9)	0.488–107	11 (79%)
Breastmilk: Plasma RTV ratio	Overall	41	0.081 (0.051, 0.100)	0.024–3.16	

Data are presented as median (Q1, Q3). Values below the lower limit of quantitation (LLoQ) were imputed as half the LLoQ: ATV plasma 11.7 ng/mL, ATV breastmilk 1.96 ng/mL, RTV plasma 4.9 ng/mL, RTV breastmilk 0.488 ng/mL.

a One participant had a breastmilk ATV concentration of 517 ng/mL and a plasma ATV concentration < LLoQ that was imputed as 11.7 ng/mL, resulting in a breastmilk: plasma ATV ratio of 44.2.

**Table 4:** Estimated relative infant dose (RID) of atazanavir (ATV) and ritonavir (RTV) (*N* = 14 mother–infant pairs).

Drug	Maternal dose (mg/day)	Median Breastmilk Concentration (ng/mL)	Max Breastmilk Concentration (ng/mL)	RID Median %	RID Max %
Atazanavir (ATV)	300	432	2,190	1.27%	6.43%
Ritonavir (RTV)	100	13.6	125	0.12%	1.10%

RID=(infant dose ÷ maternal dose) × 100, assuming infant milk intake of 150 mL/kg/day^30^. The commonly accepted threshold of concern is 10%.

No maternal AEs were reported, including no Grade 3 or higher AEs or serious adverse events. No infant experienced a Grade 3 or higher event or serious adverse event. Three infants (21%) experienced Grade 2 adverse events: one with viral rhinitis (16.6 weeks), one with a chest abscess (7.4 weeks), and one with pruritus and papular rash (4.9 weeks). None of the reported events were considered related to ATV or RTV exposure via breastmilk. Among the 14 infants, 13 (93%) tested negative for HIV during follow up, while 1 (7%) infant could not be assigned a definitive HIV status due to lack of available test results.

## Discussion

This study provides some of the first critical insights into the breastmilk transfer of ATV and RTV during breastfeeding in women living with HIV. Maternal ATV and RTV plasma exposures were generally within reported ranges in postpartum women from separate studies (31). Breastmilk-to-plasma ratios of ATV and RTV were 0.253 and 0.081, respectively, and infant plasma concentrations of both drugs were below the LLoQ at all timepoints. These findings suggest low concentrations of ATV and RTV in breastmilk, with likely even lower systemic infant exposure following oral absorption. The calculated RID values were also low, with median exposures of 1.27% for ATV and 0.12% for RTV (maximum values 6.43% and 1.10%, respectively), all below the commonly cited 10% safety threshold (30).

Maternal ATV and RTV plasma exposures were generally within reported ranges in postpartum women from separate studies, although direct comparison across studies is limited by differences in sampling design and time after dose. In the present study, maternal plasma, breastmilk, and infant plasma samples were collected at protocol-defined postpartum visits rather than as intensive pharmacokinetic profiles. Therefore, these data describe observed concentrations at the sampling times and were not designed to assess pharmacokinetic parameters such as Cmax, Tmax, or AUC. Infant samples were time-matched to maternal samples but were not collected in relation to the timing of breastfeeding; therefore, transient infant peak concentrations at unsampled times cannot be excluded. Interpretation of infant plasma concentrations below the LLoQ necessitates consideration of the dynamic nature of breastmilk drug exposure, including variability in breastmilk composition, timing of maternal dosing, timing of infant feeding, and infant absorption. These factors may contribute to variability in infant systemic exposure and may have resulted in concentrations below the assay LLoQ at the sampled timepoints. However, ATV and RTV were below the LLoQ in all 42 infant plasma samples collected across three postpartum visits, despite measurable maternal plasma and breastmilk concentrations. Together with low breastmilk-to-plasma ratios and low estimated RIDs, these findings support limited measurable systemic infant exposure to ATV and RTV from breastfeeding.

Our findings regarding ATV are novel, with the Drug and Lactation Database sponsored by the United States National Institutes of Health noting the absence of relevant published data on infant exposure to ATV via breastmilk as of October 2024 (32). Although ATV/r is no longer a preferred perinatal regimen in many settings, these findings remain clinically relevant given ongoing global use of protease inhibitor–based ART and the limited available lactation PK data for these regimens. An intensive PK study of RTV was conducted in the Malawi-based Breastfeeding, Antiretrovirals, and Nutrition (BAN) study using maternal plasma, breastmilk, and infant plasma samples (33). In the BAN study, mothers initiating a RTV boosted lopinavir (LPV)-based ART regimen at delivery with each tablet containing 50 mg of RTV and 200 mg of LPV. Dosing of this fixed dose combination tablet required two tablets to be taken twice daily for a total daily dose of RTV boosted LPV of 200/800 mg. At 6, 12, and 24 weeks, 10 mother-infant dyads contributed specimens drawn prior to dosing of RTV and LPV and then 2, 4 and 6 h after dosing, allowing for calculation of AUC_0–6_. Median and interquartile ranges (IQR) of RTV AUC_0–6hr_ were 2.30 h*µg/mL (IQR 1.76, 2.98) in maternal plasma and 0.49 h*µg/mL (IQR 0.293, 0.678), but undetectable in infant plasma, although the LLoQ was not reported in this study (33). Despite differences in PK methodology, both studies demonstrated substantially lower RTV concentrations in breastmilk relative to maternal plasma concentrations and undetectable infant plasma concentrations.

Safety data in this small cohort were favorable, with no maternal AEs reported. Three infants experienced DAIDS Grade 2 events (viral rhinitis, abscess, and pruritus with papular rash), none of which were attributed to drug exposure. No DAIDS Grade ≥3 or serious adverse events occurred. These preliminary findings align with prior lactation studies of maternal ART, which generally report low rates of drug-related toxicities in breastfed infants. Similar patterns have been described for other ARVs, including dolutegravir (34) and other protease inhibitor–based regimens (6), both of which found minimal infant adverse effects. Together, these results contribute to the limited but growing evidence on ART safety in infants breastfed by women living with HIV on ART and may help inform shared decision-making between parents and providers. Given the small size of our study and ongoing use of RTV boosted ATV as part of an ART regimen, larger studies would be valuable in validating our findings.

Recent clinical observations have shown that infant exposure to antiretroviral therapy via breastmilk can vary substantially by agent. In a retrospective cohort of eight HIV-exposed uninfected infants, two infants had serum concentrations of rilpivirine and dolutegravir above adult target ranges, and one infant's rilpivirine levels exceeded the target relative infant dose by over 600%, which was associated with clinical cessation of breastfeeding in that case (35). These findings highlight the importance of drug-specific pharmacokinetic evaluation when counseling women with HIV about breastfeeding, and contrast with the low atazanavir/ritonavir exposures observed in IMPAACT 2026.

As an opportunistic PK study, this analysis has some limitations. Since participants must have been receiving a stable RTV-boosted ATV dose for at least two weeks prior to PK sampling, the study population excluded women who started the drug but discontinued it within two weeks due to toxicity, intolerance, virologic failure, or any other reason. Thus, this study cannot identify maternal safety or tolerance issues that occur very soon after drug initiation and estimates of the frequency of adverse outcomes may understate actual maternal safety events. The modest sample size limited the ability to fully characterize interparticipant variability in maternal and infant drug exposure across postpartum timepoints and feeding practices. Variability in maternal breastmilk and plasma drug concentrations may reflect differences in the timing of maternal dosing relative to PK sampling after each dose and between study visits, potentially contributing to interparticipant and intraparticipant differences in measured drug concentrations. Infant drug concentrations were limited to plasma only; intracellular or tissue-level drug exposure was not assessed and may be more relevant for antiviral activity or toxicity. Nevertheless, the consistently low breastmilk concentrations, low RID values, and undetectable infant plasma concentrations observed across postpartum visits support limited systemic infant exposure to ATV and RTV in this cohort.

Although maternal breastmilk and infant plasma concentrations were evaluated, the study was not designed to assess HIV transmission outcomes or long-term infant clinical outcomes. Maternal HIV viral load, a key factor in assessing transmission risk, was not monitored in the IMPAACT 2026 breastfeeding component. Infant HIV infection status was assessed according to local standard-of-care procedures during study participation, but detailed infant HIV testing data and extended longitudinal follow-up beyond the study period were not available for this analysis. Infant feeding practices were documented, but the study was not designed or powered to evaluate differences in PK exposure, safety outcomes, or HIV transmission risk between exclusive breastfeeding and mixed feeding practices. Despite these limitations, these findings contribute important drug-specific lactation PK data that may help inform counseling and shared decision-making for individuals living with HIV considering breastfeeding while receiving ART. This is consistent with calls for greater research attention to antiretroviral drug transfer, safety, and HIV transmission risk in the context of breastfeeding, particularly in high-resource settings (4, 19, 36).

Although recommendations have evolved in high-resource settings to support the option of breastfeeding among people with HIV who have achieved sustained HIV-1 viral suppression, PK studies such as this one are needed for all antiretroviral medications used by breastfeeding parents, both those living with HIV and those taking HIV pre-exposure prophylaxis (27, 28, 37).

## Conclusion

ATV and RTV were detectable in breastmilk but infant plasma concentrations remained below the lower limit of quantitation at all study visits, suggesting limited measurable infant systemic exposure in this cohort. These findings add to the limited available lactation pharmacokinetic data for protease inhibitor–based ART regimens and may help inform counseling and decision-making for individuals with HIV considering breastfeeding while receiving ART.

## Data Availability

The data cannot be made publicly available due to the ethical restrictions in the study's informed consent documents and in the International Maternal Pediatric Adolescent AIDS Clinical Trials (IMPAACT) Network's approved human subjects protection plan; public availability may compromise participant confidentiality. However, data are available to all interested researchers upon request to the IMPAACT Statistical and Data Management Center's data access committee (email address: sdac.data@sdac.harvard.edu) with the agreement of the IMPAACT Network.
